# Disturbance of Plasma Lipid Metabolic Profile in Guillain-Barre Syndrome

**DOI:** 10.1038/s41598-017-08338-7

**Published:** 2017-08-15

**Authors:** Hsiang-Yu Tang, Daniel Tsun-yee Chiu, Jui-Fen Lin, Cheng-Yu Huang, Kuo-Hsuan Chang, Rong-Kuo Lyu, Long-Sun Ro, Hung-Chou Kuo, Mei-Ling Cheng, Chiung-Mei Chen

**Affiliations:** 1 Metabolomics Core Laboratory, Healthy Aging Research Center, Chang Gung University, Taoyuan, Taiwan; 2 Department of Medical Biotechnology and Laboratory Science, College of Medicine, Chang Gung University, Taoyuan, Taiwan; 3 Pediatric Hematology/Oncology, Linkou Chang Gung Memorial Hospital, Taoyuan, Taiwan; 4 Department of Neurology, Chang Gung Memorial Hospital Linkou Medical Center and College of Medicine, Chang Gung University, Taoyuan, Taiwan; 5 Clinical Phenome Center, Chang Gung Memorial Hospital, Taoyuan, Taiwan; 6 Department of Biomedical Sciences, College of Medicine, Chang Gung University, Taoyuan, Taiwan

## Abstract

Guillain-Barre Syndrome (GBS) is an inflammatory disease of the peripheral nervous system. Given that plasma metabolic profiles in GBS patients have never been explored, plasma samples of 38 GBS patients, 22 multiple sclerosis (MS) patients, and 40 healthy controls were analyzed by using untargeted and targeted metabolomics analysis. The untargeted analysis showed that levels of a set of plasma lipid metabolites were significantly decreased in GBS patients compared to the controls. Furthermore, the targeted analysis demonstrated that levels of 41 metabolites in GBS patients were significantly changed compared to either the controls or MS patients. A further metabolic analysis showed that 12 of 41 metabolites were significantly lower in classical GBS patients compared to Miller-Fisher syndrome. Among them, each of PCae C34:0, PCae C42:2, PCae C42:3, and SM C24:0 was inversely correlated with Hughes functional grading scale of GBS patients at both nadir and discharge. Receiver operating characteristic curve analysis of combination of three metabolites (PCaa C42:2, PCae C36:0 and SM C24:0) showed a good discrimination between the GBS and the controls (area under curve = 0.86). This study has demonstrated disruption of lipid metabolites in GBS may be potential biomarkers to indicate disease severity and prognosis of GBS.

## Introduction

Guillain–Barré syndrome (GBS) is a potentially life-threatening post-infection disease characterized by acute inflammatory polyneuropathy in the peripheral nervous system (PNS) with symmetrical weakness of the extremities^1^, which reaches a maximum severity within 4 weeks^2^. The overall incidence of GBS is estimated between 1.1 and 1.8 per 100,000 per year^3^. Approximately 25% of patients develop respiratory insufficiency requiring artificial ventilation^4^.

GBS is diagnosed mainly based on the clinical features of acute motor–sensory polyneuropathy with electrophysiological evidence of demyelination and/or axonal degeneration, but the abnormalities of nerve conduction velocity tend to peak at more than 2 weeks after the onset of weakness^5^. This hampers rapid diagnosis of the disease. The raised concentration of protein with a normal cell count in cerebrospinal fluid (CSF) (termed albuminocytological dissociation) is considered a hallmark of GBS^6^. This CSF picture can be seen in only about 80% of patients with GBS^7^ and in less than 10% of patients in one week after onset of the disease^8^. Most of GBS patients have a complete recovery, but up to 20% of patients remain severely disabled or die^9^. GBS is divided into different subtypes. The most common subtype is acute inflammatory demyelinating polyradiculoneuropathy (AIDP). Other subtypes involving predominantly axons are acute motor axonal neuropathy (AMAN) and acute motor and sensory axonal neuropathy (AMSAN)^10^. Miller-Fisher’s syndrome (MFS), a variant of GBS, has triad of ophthalmoplegia, ataxia, and areflexia^11^.

Although most cases of GBS (60%~70% of patients) are thought to result from an aberrant immune response triggered by a recent infectious disease^12^, the detailed molecular mechanism leading to this disease remains to be elucidated. GBS typically occurs after an infection with a pathogen that contains lipooligosaccharides that mimic the carbohydrate moiety of gangliosides present in human peripheral nerves^13^. The immune response generates antibodies that cross-react with gangliosides or ganglioside complexes which are located at specialized microdomains or lipid rafts in nerve membranes^14^. This autoimmune response results in nerve damage or functional blockade of nerve conduction. Proteomic analysis has shown several protein molecules including haptoglobin, prostaglandin D2 synthase, transthyretin and apolipoprotein A-IV that are differentially expressed in CSF of GBS patients^15^. Microarray analysis of peripheral blood reveals an altered expression of genes involved in the inflammatory response, infectious disease, cell death, hematological function, and immune cell trafficking in this disease^16^. Several other GBS-associated biomarkers including interleukin (IL)-17 and IL-22^17^, soluble C5b-9 complex complement^18^, matrix metalloproteinase-9^16^ and neurofilaments^19^ have been identified in blood samples. However, plasma metabolic profiles in GBS patients have not yet been investigated.

Metabolomics is the measurement of a large number of low-molecular-weight molecules in sample within a particular sample type by untargeted and targeted approach to provide insight into the mechanisms that underlie various physiological conditions^20^. The untargeted approach is an analysis to detect novel entities of sample, and targeted approach is a hypothesis-driven analysis that focus on specific molecules quantification^21^. In this study, initially the metabolic profiles in the plasma of a small cohort of 14 GBS patients and 12 healthy controls were determined using the untargeted and unbiased high-throughput technique (ultra high performance liquid chromatography) followed by mass spectrometry to fingerprint molecular changes involved in the metabolism. Multiple sclerosis (MS) is typically defined as a recurrent, chronic inflammatory, and demyelinating disease that affects the central nervous system (CNS)^22^, which is unlike the GBS where demyelinating occurs in PNS. To discover specific biomarkers for GBS patients, we then included MS patients as the disease control group in targeted metabolic analysis. The plasma metabolic profiles of a larger group of 38 GBS patients were then compared to that of 22 MS patients and 40 healthy controls using targeted metabolic analysis. The altered metabolites in patients with GBS were further compared to that in patients with MFS.

## Results

### Demographics of GBS patients and healthy controls

A total of 110 participants were recruited including 38 patients with GBS, 22 patients with MS, 10 patients with MFS, and 40 healthy control subjects. The average age of GBS patients was 41.3 years (range: 16 to 73), MS patients was 34.9 years (range: 19 to 60), MFS patients was 43.2 years (range: 21 to 73), and healthy controls was 41.9 years (range: 20 to 70). There was no statistical difference in age between the studied groups, but there is an increased female proportion of the MS compared to the GBS and the control group, respectively and an increased male frequency in GBS compared to the MS (Table 1). It is not surprised, given the higher prevalence rate of MS in women than men^23^ and male predominance in GBS^24^. The Hughes Functional Grading Scale (HFGS) score of GBS patients was 3.0 ± 1.2 at nadir and 2.0 ± 1.2 at discharge. The HFGS score of MFS patients was 2.0 ± 0.7 at nadir and 1.0 ± 0.0 at discharge.

**Table 1:** Basal demographics of GBS, MS and MF patients, and healthy controls.

	Controls	GBS	MS	MFS
Total number (n)	40	38	22	10
Sex (male)	62.5%	63.2%	31.8%	40%
Mean age (±SD)	41.9 ± 15.5	41.3 ± 16.1	34.9 ± 11.5	43.2 ± 16.4
HFGS at nadir	−	3.0 ± 1.2	−	2.0 ± 0.7
HFGS at discharge	−	2.0 ± 1.2	−	1.0 ± 0.0
Protein in CSF (mg/dL)		116.6 ± 85.9	36.3 ± 15.1	64.9 ± 58.1
WBC in CSF (cells/μl)		2.3 ± 2.9	2.7 ± 3.9	1.3 ± 3.5

Abbreviations: GBS, Guillain-Barre syndrome; MS, Multiple sclerosis;

HFGS, Hughes functional grading scale; WBC, white blood cells.

### Untargeted metabolomics analysis revealed a clear distinction of metabolic profiles between GBS patients and the healthy controls

For untargeted metabolomics analysis, 14 GBS patients and 12 age-matched healthy controls were recruited to examine their global metabolic profiles. Metabolic profiles demonstrated an unambiguous class separation between these two groups by principal components analysis (PCA) (Supplementary Fig. S1A). The metabolites revealed by our protocol are displayed in Supplementary Fig. S1B, and lysophosphatidylcholines (lysoPCs) and acylcarnitines (ACs) were significantly changed in GBS patients (Supplementary Fig. S1C). Except from lipid metabolites, we also found significantly increased glucose, adenine, pyroglutamate, hypoxanthine, and creatine levels in the GBS patients compared to the healthy controls (Supplementary Fig. S1D).

### Disturbances of specific lipid metabolites distinguished GBS patients from MS and MFS patients and predicted disease severity

**Figure 1:**
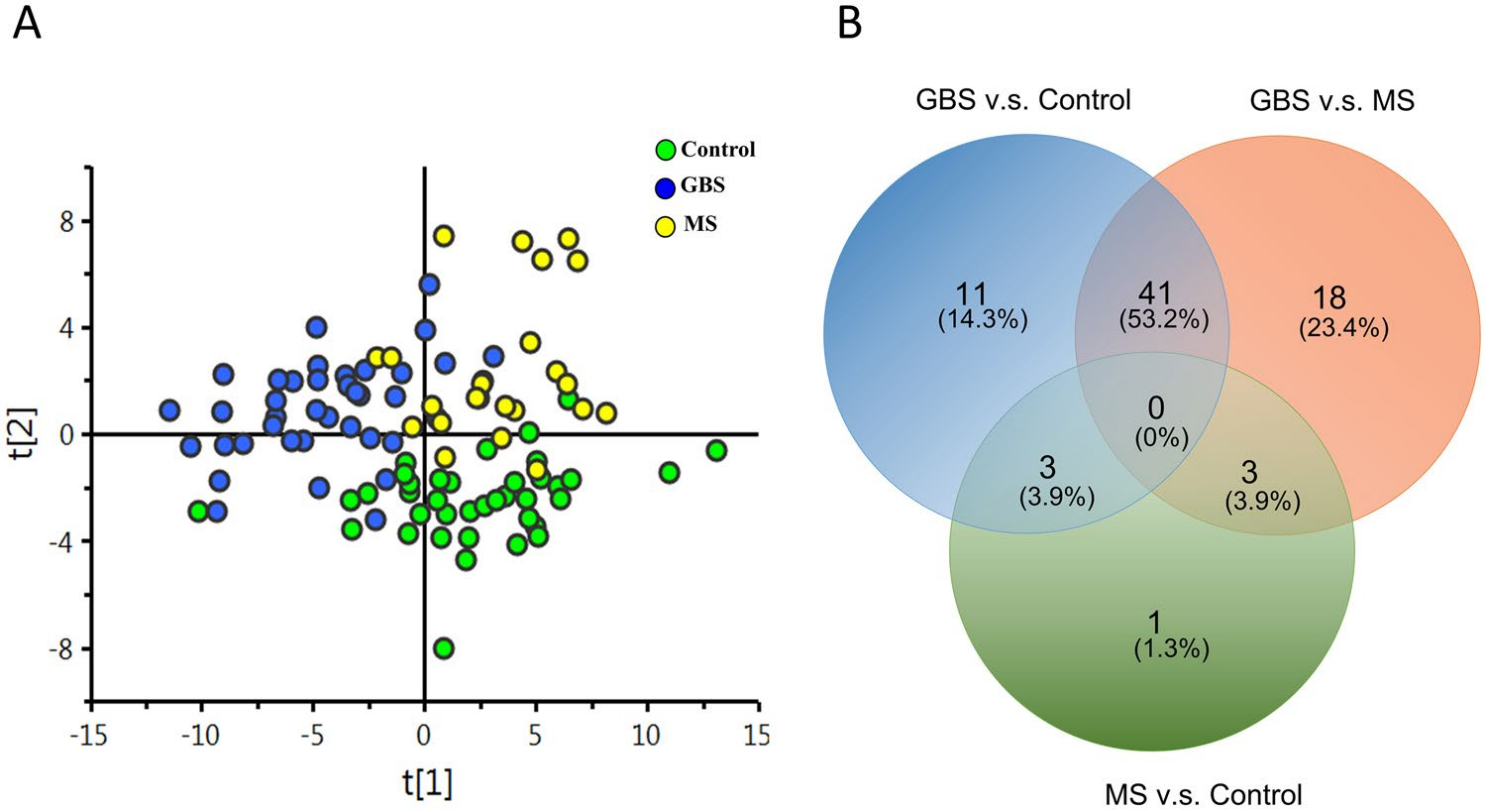
Targeted metabolomics profiles in the GBS patients, the multiple sclerosis (MS) patients, and the healthy controls (control). Extracted plasma from GBS patients (n = 38), MS patients (n = 22) and healthy controls (n = 40) were analyzed by LC-MS/MS and FIA-MS/MS in electrospray positive and negative ion mode. (**A**) The orthogonal partial least squares discriminant analysis (OPLS-DA) demonstrates a clear separation of metabolites between GBS, MS, and control cases (R2 = 0.503, Q2 = 0.338). (**B**) A Venn diagram was used to visualize the number of extremely different metabolites (p < 0.05) after one-way ANOVA analysis with Tukey’s post hoc test with false discovery rate (FDR) correction between the GBS and MS patients and the controls.

The untargeted metabolic analysis revealed that lysoPCs and ACs were potent discriminators of the GBS patients and the healthy controls. However, disadvantage of our untargeted analysis is that not all the metabolites are under the optimal extraction condition and may miss some low levels of metabolites by a combined extraction. Since most of the identified metabolites by untargeted approach belong to categories (glycerophospholipids, sphingolipids, and acylcarnitines) in AbsoluteIDQ® p180 Kit, we then used AbsoluteIDQ® p180 Kit to analyse metabolites in a larger cohort to further improve extraction method for specific metabolites. To examine whether the metabolites could discriminate GBS patients from health controls and MS patients, plasma from 38 GBS patients (including previous 14 GBS), 40 health controls (including previous 12 controls), and 22 age-matched MS patients were included for targeted metabolites quantification. We performed stable isotope dilution-multiple reaction monitoring mass spectrometry to identify and quantify the plasma level of glycerophospholipids, sphingolipids, ACs, amino acids, and biogenic amines. A total of 183 metabolites where concentrations were below the limit of detection (<LOD) or below lower limit of quantification (<LLOQ) were excluded, and remaining 117 metabolites with good quality were included for analysis (Supplementary Table 1). The principal component analysis (PCA) (Supplementary Fig. S2) and orthogonal partial least squares discriminant analysis (OPLS-DA) result (Fig. 1A) of 117 available metabolites from targeted analysis demonstrated a clear separation of the metabolite profile among GBS, healthy controls, and MS patients. This OPLS-DA result indicated a specific GBS signature. A total of 63 metabolites were significantly different between GBS patients and controls, and 55 metabolites remained significantly different after false discovery rate (FDR) adjustment (Supplementary Table 1). Among these metabolites, were significantly lower plasma levels of aspartate, creatinine, serotonin, taurine, phosphatidylcholines (PCs), lysoPCs, sphingomyelins (SMs), and ACs in the GBS patients compared to the controls and significantly higher plasma levels of isoleucine in the GBS patients compared to the controls.

**Figure 2:**
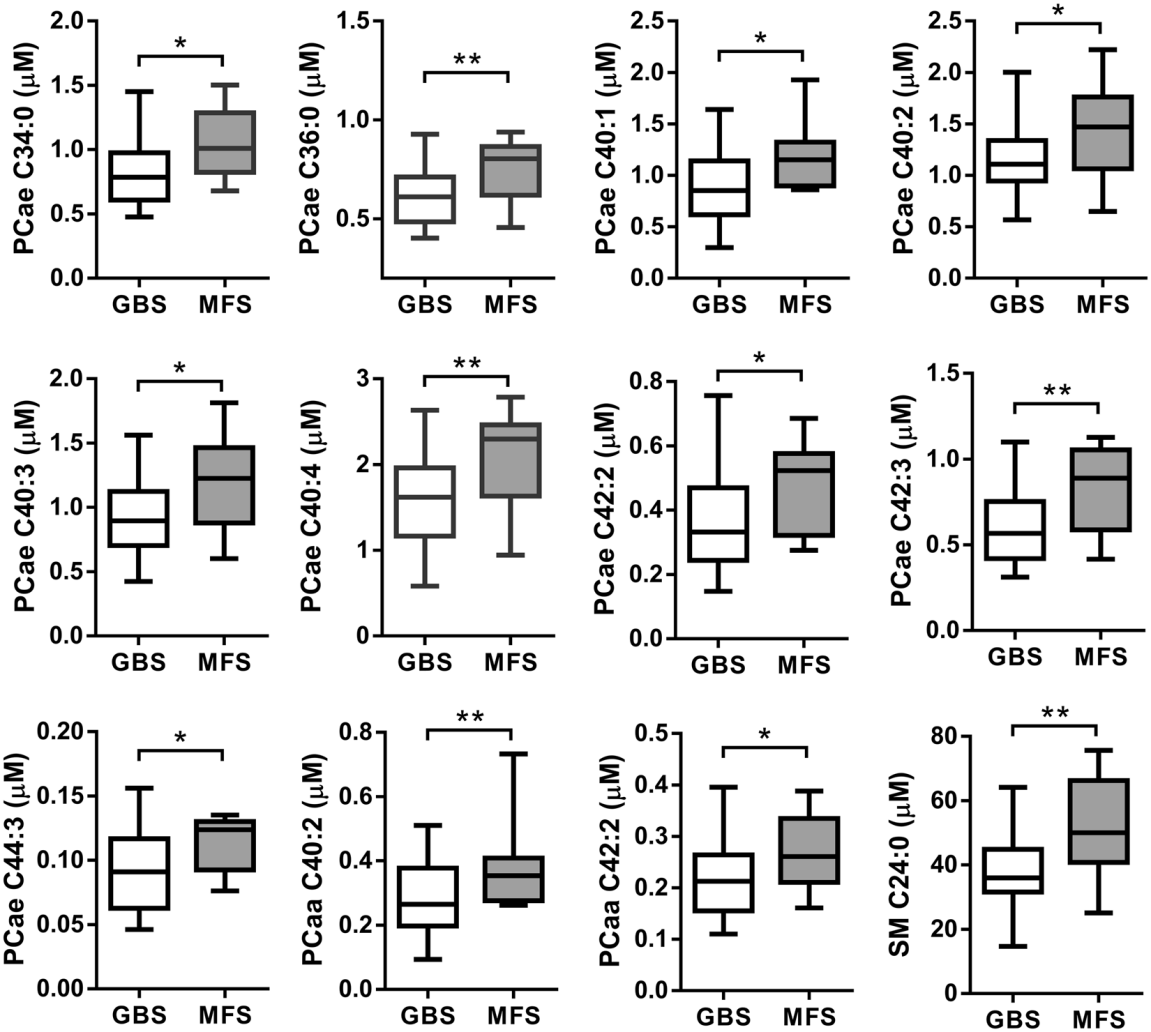
Metabolites were significantly decreased in GBS patients (n = 38) compared to Miller Fisher syndrome (MFS) (n = 10) patients. Statistical differences were determined by Student t-test; *p < 0.05, **p < 0.01.

**Figure 3:**
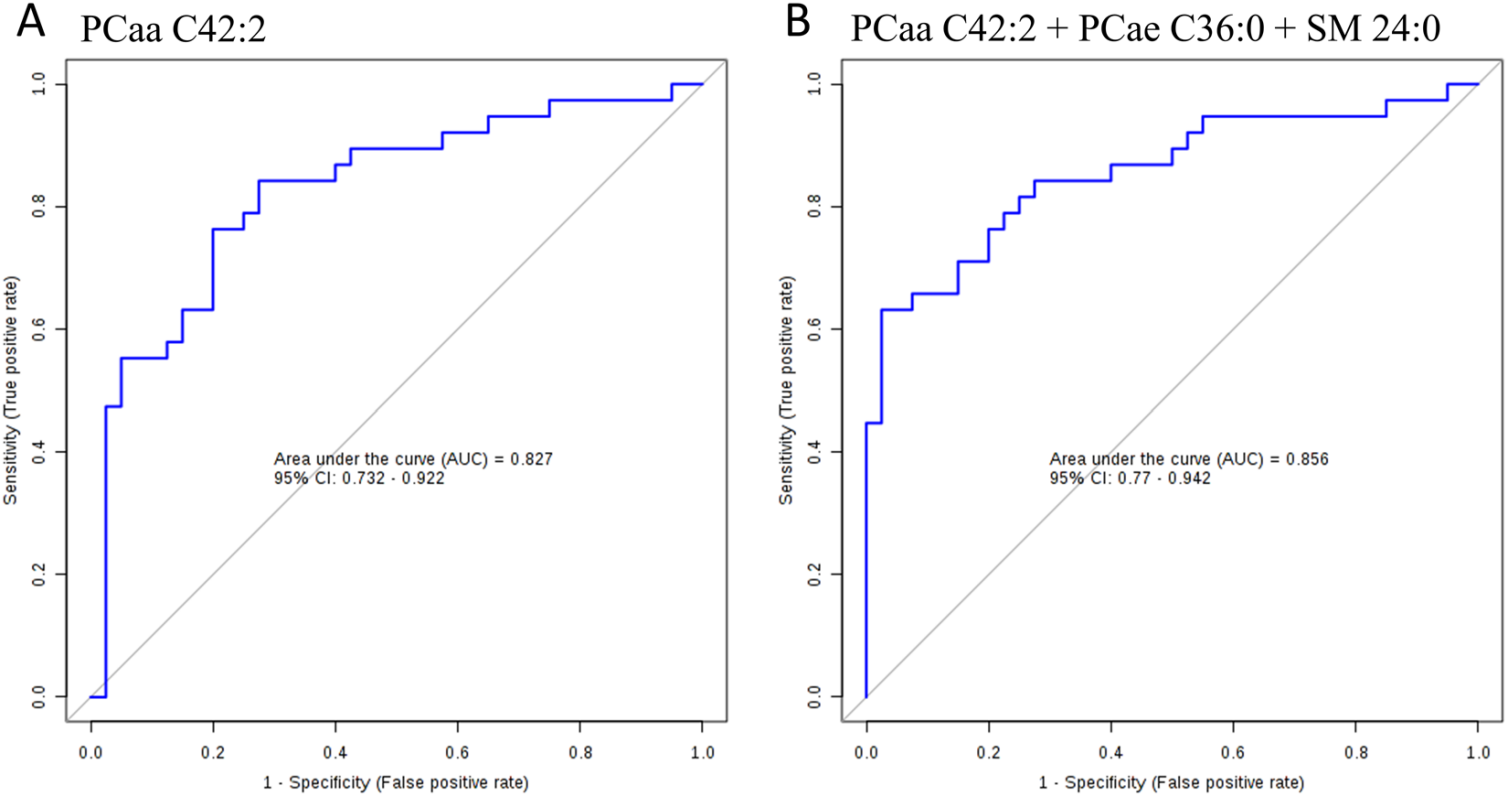
Receiver operating characteristics (ROC) curves analysis. ROC curve models of (**A**) PCaa C24:2 and (**B**) combined metabolites of PCaa C42:2 + PCae C36:0 + SM 24:0 to discriminate GBS patients from the controls.

A venn diagram was used to visualize the number of extremely different metabolites (p < 0.05) between GBS patients, controls, and MS patients (Fig. 1B). The result showed that 40 metabolites comprising 11 PC diacyl (aa), 19 PC acyl-alkyl (ae), 4 hydroxysphingomyelin (SM(OH)), 4 SMs, lysoPC C18:2, and AC 14:1 were significantly depleted in the plasma of GBS patients compared to the healthy controls and the MS patients, and only isoleucine was significantly increased in GBS patients compared to the healthy controls and MS patients, respectively (Table 2). These data implied that the 41 metabolites could be used to distinguish GBS (acute peripheral demyelinating) from not only the controls but also acute stage of MS (central demyelinating). In order to delineate whether changes of these metabolites could help to separate the classic GBS and MFS (the subtype of GBS), we then compared the identified 41 metabolites between GBS and MFS. The results showed that the levels of PCae C34:0, PCae C36:0, PCae C40:1, PCae C40:2, PCae C40:3, PCae C40:4, PCae C42:2, PCae C42:3, PCae C44:3, PCaa C40:2, PCaa C42:2, and SM C24:0 were significantly lower in classical GBS compared to MFS (Fig. 2). The majority of these metabolites were long chain fatty acid-containing phosphatidylcholines and SM (carbon number of fatty acid > 18), indicating that specific lipids disturbances occurred in GBS patients. Among the 12 metabolites, 9 metabolites were inversely correlated with the HFGS score at nadir, revealing the lower lipid level, the higher disease severity (Table 3). Furthermore, each of PCae C34:0, PCae C42:2, PCae C42:3, and SM C24:0 was inversely correlated with the HFGS score at discharge, suggesting the lower lipid level, the worse prognosis (Table 3). The receiver operating characteristics (ROC) curves and area under curve (AUC) values of each of the 12 metabolites (Supplementary Table 2) revealed that PCaa C42:2 could predict GBS with 0.83 AUC, 0.76 sensitivity, and 0.80 specificity (Fig. 3A). ROC curves of different combinations from 12 metabolites showed that the combination of PCaa C42:2, PCae C36:0, and SM C24:0 allows a good prediction for GBS (AUC = 0.86) with 0.79 sensitivity and 0.78 specificity (Fig. 3B).

**Table 2:** Levels of significantly changed metabolites in GBS patients compared to healthy controls and MS patients revealed by targeted metabolomics analysis.

	Metabolites (μM)	Control (mean ± sd)	GBS (mean ± sd)	MS (mean ± sd)	p value^a^ (GBS/Control)	p value^a^ (GBS/MS)	p value^a^ (MS/Control)
1	AC14:1	0.11 ± 0.03	0.08 ± 0.02	0.11 ± 0.03	<0.01	<0.01	1.170
2	Isoleucine	88.41 ± 25.22	109.93 ± 37.40	84.65 ± 18.65	0.013	0.012	1.119
3	lysoPCa C18:2	48.20 ± 15.20	39.09 ± 15.22	50.51 ± 13.85	0.048	0.031	1.156
4	PCaa C28:1	1.62 ± 0.40	1.26 ± 0.45	1.58 ± 0.43	<0.01	0.034	1.098
5	PCaa C32:3	0.28 ± 0.05	0.22 ± 0.08	0.31 ± 0.08	<0.01	<0.01	0.902
6	PCaa C34:4	1.10 ± 0.35	0.82 ± 0.47	1.14 ± 0.44	0.029	0.028	1.093
7	PCaa C36:0	3.64 ± 1.10	2.36 ± 0.81	3.81 ± 0.96	<0.01	<0.01	1.161
8	PCaa C36:6	0.58 ± 0.20	0.41 ± 0.22	0.65 ± 0.28	<0.01	<0.01	0.965
9	PCaa C38:0	3.35 ± 0.91	2.61 ± 0.80	3.62 ± 0.85	<0.01	<0.01	0.981
10	PCaa C40:2	0.42 ± 0.11	0.28 ± 0.10	0.40 ± 0.09	<0.01	<0.01	1.164
11	PCaa C40:3	0.58 ± 0.13	0.43 ± 0.13	0.56 ± 0.13	<0.01	<0.01	1.164
12	PCaa C42:2	0.32 ± 0.08	0.22 ± 0.07	0.30 ± 0.06	<0.01	<0.01	1.065
13	PCaa C42:4	0.23 ± 0.05	0.17 ± 0.06	0.22 ± 0.06	<0.01	<0.01	1.165
14	PCaa C42:5	0.33 ± 0.09	0.24 ± 0.09	0.35 ± 0.21	0.010	0.011	1.120
15	PCae C32:2	0.45 ± 0.12	0.34 ± 0.10	0.50 ± 0.13	<0.01	<0.01	0.692
16	PCae C34:0	1.01 ± 0.25	0.83 ± 0.25	1.11 ± 0.27	0.015	<0.01	0.908
17	PCae C34:2	9.01 ± 2.49	6.61 ± 2.48	9.37 ± 2.82	<0.01	<0.01	1.185
18	PCae C34:3	6.66 ± 1.99	4.09 ± 1.88	6.18 ± 2.36	<0.01	<0.01	1.083
19	PCae C36:0	0.80 ± 0.17	0.61 ± 0.13	0.82 ± 0.14	<0.01	<0.01	1.176
20	PCae C36:1	7.45 ± 1.30	6.46 ± 1.73	7.94 ± 1.63	0.036	<0.01	0.991
21	PCae C36:3	5.67 ± 1.37	4.23 ± 1.47	5.78 ± 1.65	<0.01	<0.01	1.085
22	PCae C36:4	14.18 ± 3.61	10.95 ± 3.98	14.51 ± 4.19	<0.01	<0.01	1.107
23	PCae C36:5	10.39 ± 2.78	7.38 ± 2.59	10.78 ± 3.40	<0.01	<0.01	1.174
24	PCae C38:0	1.43 ± 0.39	1.08 ± 0.35	1.45 ± 0.41	<0.01	<0.01	1.065
25	PCae C38:3	3.75 ± 0.70	3.11 ± 1.10	3.88 ± 1.09	0.029	0.023	1.150
26	PCae C38:6	6.70 ± 1.73	4.94 ± 1.55	7.06 ± 1.50	<0.01	<0.01	1.103
27	PCae C40:1	1.19 ± 0.30	0.89 ± 0.34	1.17 ± 0.26	<0.01	<0.01	1.078
28	PCae C40:2	1.35 ± 0.26	1.15 ± 0.32	1.51 ± 0.40	0.029	<0.01	0.615
29	PCae C40:3	1.17 ± 0.26	0.91 ± 0.29	1.20 ± 0.26	<0.01	<0.01	1.163
30	PCae C40:4	1.87 ± 0.40	1.58 ± 0.51	2.09 ± 0.42	0.031	<0.01	0.597
31	PCae C42:2	0.48 ± 0.10	0.36 ± 0.15	0.53 ± 0.18	<0.01	<0.01	0.905
32	PCae C42:3	0.76 ± 0.17	0.59 ± 0.20	0.83 ± 0.17	<0.01	<0.01	0.944
33	PCae C44:3	0.11 ± 0.02	0.09 ± 0.03	0.12 ± 0.03	<0.01	<0.01	1.008
34	SM(OH) C14:1	5.74 ± 1.22	4.81 ± 1.50	6.19 ± 1.36	0.023	<0.01	0.977
35	SM(OH) C22:1	42.92 ± 8.50	31.34 ± 10.84	40.59 ± 9.53	<0.01	<0.01	1.097
36	SM(OH) C22:2	41.54 ± 7.06	31.23 ± 9.24	41.79 ± 7.20	<0.01	<0.01	1.018
37	SM(OH) C24:1	1.72 ± 0.37	1.28 ± 0.44	1.59 ± 0.42	<0.01	0.033	0.951
38	SM C16:0	182.91 ± 25.79	159.18 ± 35.26	193.46 ± 37.33	0.013	<0.01	0.985
39	SM C16:1	25.49 ± 3.76	22.11 ± 5.42	26.95 ± 6.95	0.032	<0.01	1.015
40	SM C24:0	50.88 ± 9.35	37.79 ± 12.45	47.52 ± 11.37	<0.01	0.010	0.971
41	SM C26:0	0.33 ± 0.09	0.26 ± 0.08	0.33 ± 0.09	<0.01	0.015	0.999

^a^One-way ANOVA with Tukey’s post hoc test and with FDR correction.

Abbreviations: GBS, Guillain-Barre syndrome; MS, multiple sclerosis.

**Table 3:** Spearman’s rho correlation coefficients among plasma levels of lipid metabolites and HFGS of GBS patients at nadir and at discharge.

	HFGS at nadir	HFGS at discharge
PCaa C40:2	−0.35*	−0.21
PCaa C42:2	−0.43**	−0.29
PCae C34:0	−0.39*	−0.35*
PCae C36:0	−0.24	−0.14
PCae C40:1	−0.38*	−0.28
PCae C40:2	−0.23	−0.14
PCae C40:3	−0.37*	−0.27
PCae C40:4	−0.44 **	−0.30
PCae C42:2	−0.40*	−0.36*
PCae C42:3	−0.48**	−0.40*
PCae C44:3	−0.30	−0.16
SM C24:0	−0.46**	−0.39*

*Correlation is significant at the 0.05 level.

**Correlation is significant at the 0.01 level.

## Discussion

Identification of biomarkers in aberrant biochemical pathways for GBS characterized by peripheral nerve damage is an important step toward improving our understanding of disease pathogenesis and may help to reveal potential therapeutic targets. Blood-based metabolomics analysis should be an attractive option for discovering useful biomarkers. Our targeted metabolomics analysis demonstrated that levels of 41 metabolites (including isoleucine, ACs, lysoPCs, PCs, and SMs) in GBS patients were significantly changed when compared to either the controls or MS patients. Furthermore, our study showed that a set of 12 metabolites profile featuring PCs and SMs were significantly decreased in GBS patients, which may distinguish GBS from MFS. Except from targeted metabolites analysis, untargeted metabolites screening also showed that the energy related metabolites were significantly increased in GBS patients compared to controls. As these metabolites reflecting cell membrane integrity are highly correlated with HFGS at both nadir and discharge, they should serve as useful biomarkers for indicating disease severity and prognosis of GBS.

Neurological dysfunction in GBS patients is associated with reduced conductance associated with demyelination and/or axonal loss. Unlike the CNS nerves, peripheral nerves can assume an immature cell phenotype and regenerate spontaneously after injury in a permissive environment^25^. To repair the damage of sheath during demyelination, cells require extremely high level of lipid from extracellular environment^26^. We summarize different lipid compositions that are specific for myelin and remarkably altered in GBS patients (Supplementary Fig. S3). Because plasmalogens contain the long-chain fatty acids (20 to 24 carbon atoms) and peripheral myelinated nerves are enriched in sphingolipids^26^, it is proposed that lipid metabolic defects in GBS including lysoPCs, PCs, and SMs may cause or contribute to impaired myelin regeneration. We have also found that the plasma cholesterol level in GBS was significantly decreased about 15% as compared to that of health control (Supplementary Fig. S4), and MS patients also showed 17% decrease of cholesterol as compared to that of health controls. Cholesterol is essential for integrating myelin component and the myelin compaction depends on cholesterol level in Schwann cells of PNS^27, 28^.

Another support of disturbed lipid metabolism in GBS patients is the significant decrease of ACs in plasma of these patients. ACs play a major role in central carbon and lipid metabolism occurring in mitochondria^29^. ACs and carnitine supplementation produce beneficial effects in the treatments of various neurological diseases^30^. ACs play multifactorial roles for neuroprotection^31^ including improvement of mitochondrial energetics and function, antioxidant activity, and stabilization of membranes^32, 33^. ACs have neuromodulatory action by increasing the synthesis of phospholipids required for membrane formation and integrity^34^. The significant decrease of ACs in GBS patients suggests disturbed lipid metabolism, which may contribute to demyelination in GBS. However, the cause of decreased ACs in GBS patients remains to be elucidated.

Abnormal energy metabolism could be an underlying mechanism linking disturbed lipid metabolism and axon integrity because myelin could produce adenosine triphosphate (ATP) to support the axon energy demand by glycolysis and Krebs cycle^35, 36^ to maintain structural integrity of axons. Metabolic imbalances that occur in neuron diseases affecting axonal integrity are particularly significant, given the broad association between energy metabolism and axonal damage^37^. Several models of inflammation-induced demyelination show spontaneous remyelination and repairment in inactive lesions as well as in lesions with ongoing demyelinating activity^38–41^, in which the demand of energy is increased by increasing mitochondrial density in demyelinated axons^35, 42^. Recently, substantial evidence has shown that increased mitochondrial metabolism and lipogenesis are necessary for normal myelination during Schwann cell differentiation and mitochondrial metabolism dysfunctions would lead to hypomyelination^43^. Adenine is required to form ATP and nicotinamide adenine dinucleotide (NAD). ATP and NAD are important coenzymes for energy production and cell survival. The high plasma levels of glucose, adenine and hypoxanthine in GBS indicating high activity of energy metabolism in cells may reflect a compensatory response to demyelination. The creatine/phosphocreatine shuttle system generates ATP much faster than the glycolysis and oxidative phosphorylation, which plays a pivotal role in muscle and nervous system. The increased level of creatine in GBS patients also indicates the increased energy requirement during demyelination and remyelination.

Increased level of isoleucine in plasma of GBS patients is another indicator of abnormal energy metabolism, and such increase can enhance glucose uptake and whole body glucose oxidation^44^. The isoleucine could provide substrates like acetyl-CoA and succinyl-CoA to tricaboxylic acid (TCA) to produce ATP. It also can provide the carbon skeletons for glutamate and glutamine synthesis as role of detoxification^45^. Additionally, isoleucine is associated with the G-protein coupled receptor and ERK signaling pathways in intestinal epithelia^46^, and is required for lymphocytes growth and proliferation^47^. The increased level of isoleucine in GBS patient may play an essential role in regulating energy metabolism and immunity reaction.

Increased pyroglutamate, a lactam of glutamic acid, is another contribuing factor to the abnormal plasma metabolism in GBS patients. It has been found as an N-terminal modification in many neuronal peptides playing as a role in activity and stability^48^. The level of pyroglutamate increased in GBS patients could come from glutathione degradation, proteins degradation, and glutamate reaction^49^. In neuronal cell, pyroglutamate is an intermediate to regulate glutamate reservoir when glutathione rapid turns over^50^. Several studies have reported that accumulation of pyroglutamate-containing peptides increased toxicity in Alzheimer’s disease^51^, however the mechanism and the role of elevated level of pyroglutamate in GBS patients should be investigated further in the future.

Although our study is the first one using untargeted and targeted metabolomics to investigate plasma metabolic profiles in GBS, this study has several limitations. Firstly, the sample sizes of the study groups may not be large enough to detect smaller changes of metabolites in GBS. Secondly, because of higher prevalence of female than male in MS, the MS group composed of more female patients compared to the GBS and the controls may cause some bias results contributed by gender effects on the metabolites. Thirdly, we used combined extractions for global screening as suggested by Wishart^52^, but a recent study has shown that to analyse the aqueous and organic extracts separately may be better than combining extractions to wide the metabolome coverage^53^. Fourthly, we did not include a group of patients with other acute peripheral neuropathy for comparisons, which may serve as a better disease control than MS.

**Figure 4:**
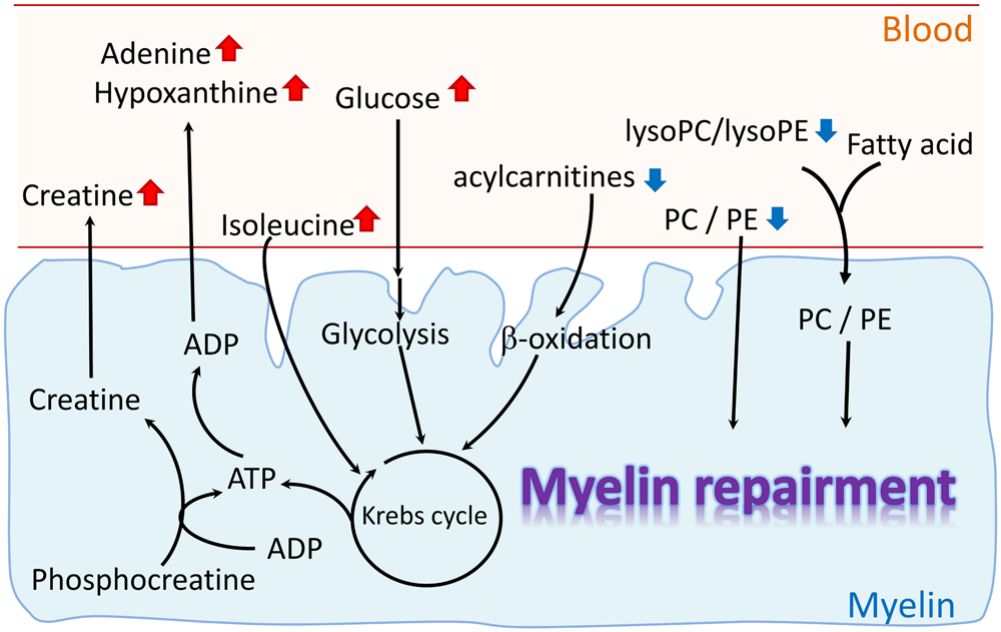
A simple diagram to illustrate the lipid metabolites disturbance in GBS patients. Decreased plasma levels of PCs, lysoPCs/lysoPEs reveal that myelin cells may recruit those lipids to myeline repairment. And the increased plasma levels of adenine, hypoxanthine, creatine, and glucose react to the energy demand for myelin repairmen.

Based on our finding, we propose a model to explain the changes of metabolites in GBS patients (Fig. 4). During the demyelination, the myelin may have spontaneous remyelination and repair in lesions with ongoing demyelinating activity. The decreased PCs, lysoPCs, and SMs levels in plasma implicate the demand of substances for membrane repair, and the increased adenine and hypoxanthine reveals increased energy requirement simultaneously. In summary, the results of the current study indicate that changes in metabolic profiles should be helpful in clinical diagnosis of differentiating typical GBS from not only controls, but also MS and MFS, as well as to predict the disease progression of GBS. These data suggest that specific lipid disturbances are associated with demyelinating polyneuropathy in GBS patients. Lipid profile in GBS patients may serve an important function as markers to indicate impaired myelin membrane integrity in GBS patients. However, further investigations are required to clarity the causal relationship and the underlying mechanism to consolidate our findings. Furthermore, a study including a larger series of patients will be warranted to consolidate our results. The altered metabolites may need to be examined in GBS patients at recovered stage in order to delineate the treatment effects on these metabolic changes.

## Methods

### Evaluation of functional impairment in patients and sample preparation

Plasma samples of 38 GBS, 22 MS, and 10 MFS patients were collected in EDTA-containing tubes within two weeks after onset of the disease and before therapeutic interventions. Patients with GBS and MFS fulfilled the standard diagnostic criteria^39^. Based on the clinical features and the results of nerve conduction velocity study, 38 GBS patients were all classified as the AIDP subtype. MS patients were all in an acute relapsing stage and diagnosed as MS according to the McDonald’s criteria^42^. Plasma samples were collected in EDTA-containing from 40 healthy controls confirmed to have no neurological and systemic diseases including autoimmune diseases, infection and malignancies. Initially, 14 GBS patients and 12 controls were included for untargeted metabolomics analysis to globally screen the difference between GBS patients and controls. Subsequently, according to the findings of untargeted analysis, 38 GBS patients, 22 MS patients and 40 controls were performed to identify and quantify targeted metabolites using AbsoluteIDQ® p180 Kit. Finally, the 41 metabolites identified from targeted analysis were examined for comparisons between 38 GBS and 10 MFS patients. A standard case collection form was used to record age at onset, gender, clinical manifestations, and results of plasma biochemistry surveys. Motor functional deficits of GBS patients were scored at nadir and at discharge by the Hughes Functional Grading Scale (HFGS) ranging from 0 to 6^54^. Nadir of the disease was defined as the highest HFGS score. The scale was specifically defined as follows: 0, healthy state; 1, minor symptoms and capable of running; 2, able to walk 5 m or more without assistance but unable to run; 3, able to walk 5 m across an open space with help; 4, bedridden or chair bound; 5, requiring assisted ventilation for at least part of the day; 6, dead^54^. A higher HFGS score indicates a more disease severity and worse outcome. This study was performed under a protocol approved by the Institutional Review Boards of Chang Gung Memorial Hospital, Taiwan (ethical license No: 97–2499 C; 102-2260A3). All examinations and experiments including plasma sample collection were carried out in accordance with relevant guidelines and regulations and were performed after obtaining written informed consents.

### Metabolite extraction and analysis of untargeted metabolomics profile by LC-TOF/MS

The extractive method has been described by David Wishart^52^. In briefly, to 100 μl plasma, 400 μl acetonitrile with 0.1% formic acid containing 100 ng/ml debrisoquine (Sigma Aldrich, USA) as an internal control was added. The mixture was vortexed for 30 seconds at 4 °C, sonicated for 15 min on ice bath for good mixing, and centrifuged at 12,000 g for 30 min to remove the precipitates. The supernatant was collected into a separate microcentrifuge tube. The pellet was re-extracted once. To the residual pellets, 400 μl of 50% methanol with 0.1% formic acid was added. The suspension was vortexed for 30 seconds, and was sonicated for 15 min and again was centrifuged to remove the precipitates. The aqueous methanolic supernatant and acetonitrile supernatant were pooled and dried under nitrogen gas. Prior to analysis, the sample was dissolved in 100 μl of 95:5 water/acetonitrile containing 0.1% formic acid and centrifuged at 12,000 g for 30 min. The clear supernatant was collected for LC-TOF/MS analysis. The procedure was carried out by the method of Cheng *et al*.^55^. Briefly, each sample was separated with a 100 mm × 2.1 mm, 1.7 μm C8 column (Waters Corp., Milford, USA) at 45 °C using an ACQUITY TM UPLC system (Waters Corp., Milford, USA). Samples were eluted from LC column at a flow rate of 0.5 ml/min using a linear gradient: 0–1.25 min: 1–50% B; 1.25–2.5 min: 50–99% B; 2.5–4.5 min: 99% B; 4.5–6.6 min: 1% B for re-equilibration. The mobile phases were 0.1% formic acid (solvent A) in water and 0.1% formic acid in acetonitrile (solvent B). The eluent was introduced into the TOF MS system (SYNAPT G1, Waters Corp., Milford, USA) and operated in an ESI positive ion mode. The desolvation gas was set to 700 l/h at a temperature of 300 °C, cone gas set to 25 l/h, and source temperature set at 80 °C. The capillary voltage and cone voltage were set to 3,000 V and 35 V, respectively. The MCP detector voltage was set to 1,700 V. The data acquisition rate was set at 0.1 s with a 0.02 s interscan delay. The data were acquired in centroid mode from 20 to 990 m/z. For accurate mass acquisition, a lock-mass of sulfadimethoxine at a concentration of 60 ng/ml and a flow rate of 6 μl/min (an [M + H]+ ion at 311.0814 Da). Sample order was randomized during analysis. To monitor the data quality, we analyzed the sample queue for six times to confirm the centralization of six replicates for each sample (Supplementary Table 3 and Supplementary Fig. S5). Findings and alignments of metabolites were executed by MarkerLynx (Waters, Milford, USA) (Supplementary method).

To confirm the identification of untargeted metabolites, tandem mass spectra from samples was then compared to standards under identical chromatographic conditions with those of the profiling experiment or compared to mass spectra of model compounds from databases of METLIN (https://metlin.scripps.edu/index.php) and HMDB (http://www.hmdb.ca/).

### Identification and quantification of targeted metabolites by tandem mass (MS/MS)

The quantitative assay of metabolome were further carried out by the commercially available Biocrates AbsoluteIDQ® p180 Kit (Biocrates Life Science AG, Innsbruck, Austria), which we applied previously^56^. The kit was used to simultaneously identify and quantify 183 metabolites within 5 compound classes. These metabolites include 87 glycerophospholipids and 14 sphingolipids, 20 biogenic amines, 40 acylcarnitines, 21 amino acids, and 1 hexose. All reagents used in this analysis were LC-MS grade. Each 10 μl of plasma was prepared in line with manufacturer’s directive. Liquid chromatography coupled with tandem mass (LC-MS/MS) method was used to quantify biogenic amines and amino acids, and flow injection analysis coupled with tandem mass (FIA-MS/MS) method was used to quantify the other lipid species. The analysis was performed in positive electrospray ionization mode with multiple reaction monitoring (Waters, Milford, USA). For LC-MS/MS analysis, chromatographic separation was performed on an Acquity BEH C8 column (75 mm × 2.1 mm, particle size of 1.7 μm) (Waters, Milford, USA) at 50 °C using a gradient mixture of solvent A (water with 0.2% formic acid) and solvent B (acetonitrile with 0.2% formic acid) at a flow rate of 0.9 ml/min using a linear gradient: 0–0.38 min: 0% B; 0.38–3 min: 0–15% B; 3–5.4 min: 15–70% B; 5.4–5.93 min: 100% B; 5.93–6.6: 0% B for re-equilibration. The optimized parameters were as following: capillary at 3.2 kV; desolvation gas flow at 1200 l/h; cone gas flow at 150 l/h; desolvation temperature at 650 °C; source temperature at 150 °C; cone voltage at 10 V, respectively. For FIA analysis, an isocratic method at 0.03 ml/min was used with commercial solvent. The parameters as following: capillary at 3.9 kV; desolvation gas flow at 650 l/h; cone gas flow at 150 l/h; desolvation temperature at 350 °C; source temperature at 150 °C; cone voltage at 20 V, respectively. Sample order was randomized and 3 different levels of quality controls run on each plate. Data were corrected between batches using the results of 3 quality controls with Metaboanalyst 3.0 (http://www.metaboanalyst.ca), and the CV of 3 quality control (QC) samples containing low, middle, and high level of targeted metabolites included in Supplementary Table 4. Targeted metabolic data were analyzed by TargetLynx (Waters, Milford, USA). MetIDQ software (Biocrates, Innsbruck, Austria) was applied to integrate the 183 targeted metabolites by automated calculation of metabolite concentrations. Metabolites where concentration were below the limit of detection (<LOD) or below lower limit of quantification (<LLOQ) were excluded. The remaining 117 metabolites with good quality composed of 72 glycerophospholipids, 14 sphingolipids, 7 acylcarnitines, 3 biogenic amines, and 21 amino acids were finally analyzed

### Statistical methods

All data analyses were performed on IBM SPSS 20.0. Student’s *t*-test, Mann-Whitney U test or one way ANOVA with Tukey’s post hoc test and with FDR correction were performed for comparisons between groups where appropriate. The Spearman’s rho correlation coefficient was used to analyze correlations between levels of metabolites and HFGS. All *P*-values were two-tailed. The values of *P* < 0.05 were considered significant. The visualization models include unsupervised PCA and OPLS-DA model were applied, and performed using the software of SIMCA-P (Umetrics AB, Umea, Sweden). The VIP value of each variable in the model was calculated to indicate its contribution to the classification. A higher VIP score was indicative of a stronger contribution to discrimination among groups. An analysis of the ROC curve was performed by metaboanalyst 3.0 to measure the ability of metabolites to predict GBS.

## Electronic supplementary material

Supplementary information
