# Supplementary material for: Disturbance of Plasma Lipid Metabolic Profile in Guillain-Barre Syndrome
Source: Sci Rep. 2017 Aug 15;7:8140. doi: 10.1038/s41598-017-08338-7 (PMC5557802; doi:10.1038/s41598-017-08338-7)
Supplement: Supplementary file 1 — Supplementary information [file 41598_2017_8338_MOESM1_ESM.doc]

Supplementary information

**Disturbance of Plasma Lipid Metabolic Profile in Guillain-Barre Syndrome**

Hsiang-Yu Tang^1^, Daniel Tsun-yee Chiu^1,2,3^, Jui-Fen Lin^1^, Cheng-Yu Huang^1^, Kuo-Hsuan Chang^4^, Rong-Kuo Lyu^4^, Long-Sun Ro^4^, Hung-Chou Kuo^4^, Mei-Ling Cheng^1,5,6,*^, Chiung-Mei Chen^4^,^*^

^1^Metabolomics Core Laboratory, Healthy Aging Research Center, Chang Gung University, Taoyuan, Taiwan.

^2^Department of Medical Biotechnology and Laboratory Science, College of Medicine, Chang Gung University, Taoyuan, Taiwan

^3^Pediatric Hematology/Oncology, Linkou Chang Gung Memorial Hospital, Taoyuan, Taiwan

^4^Department of Neurology, Chang Gung Memorial Hospital Linkou Medical Center and College of Medicine, Chang Gung University, Taoyuan, Taiwan

^5^Clinical Phenome Center, Chang Gung Memorial Hospital, Taoyuan, Taiwan

^6^Department of Biomedical Sciences, College of Medicine, Chang Gung University, Taoyuan, Taiwan

*Correspondence:

Chiung-Mei Chen, email: cmchen@adm.cgmh.org.tw,

Tel.: +886-3-3281200x8729; Fax: +886-3-3287226

and Mei-Ling Cheng, email: chengm@mail.cgu.edu.tw,

Tel. & Fax: +886-3-2118244

**Supplementary method**

Alignment with MarkerLynx

For untargeted analysis, findings and alignments of features were executed by MarkerLynx (Waters, Milford, USA). Peak detection was from retention time 0 min to 3.4 min. The collection parameters were as the following: Mass error was 0.03 Da; threshold on intensity was set as 30 counts; retention time window was 0.1 min; element compositions of features contain C, H, N, O, S, Na, P with monoisotopic mode in 5 mDa mass tolerance.

Supplementary Table 1. The 117 metabolites were chosen from AbsoluteIDQ® p180 Kit and quantified by targeted metabolomics analysis.

|  | Metabolite  (μM) | Control  (mean ± sd) | GBS  (mean ± sd) | MS  (mean ± sd) | p-value^a^  (GBS/Control) | p-value^a^  (GBS/MS) | p-value^a^  (MS/Control) |
| --- | --- | --- | --- | --- | --- | --- | --- |
| 1 | AC0 | 33.69±8.46 | 36.94±10.00 | 36.79±8.19 | 0.73 | 0.82 | 0.43 |
| 2 | AC2 | 6.52±2.52 | 6.84±4.73 | 4.93±2.08 | 0.25 | 1.00 | 0.40 |
| 3 | AC10:1 | 0.45±0.09 | 0.34±0.08 | 0.35±0.09 | < 0.01 | 0.83 | < 0.01 |
| 4 | AC10:2 | 0.20±0.05 | 0.17±0.04 | 0.17±0.03 | < 0.01 | 0.85 | 0.09 |
| 5 | AC14:1 | 0.12±0.03 | 0.09±0.02 | 0.11±0.03 | < 0.01 | < 0.01 | 0.76 |
| 6 | AC14:2 | 0.07±0.03 | 0.05±0.03 | 0.05±0.02 | < 0.01 | 0.88 | < 0.01 |
| 7 | AC18:2 | 0.09±0.04 | 0.09±0.04 | 0.08±0.05 | 0.91 | 0.11 | 0.20 |
| 8 | lysoPCa C16:0 | 154.97±32.14 | 126.41±41.28 | 141.60±27.69 | < 0.01 | 0.24 | 0.33 |
| 9 | lysoPCa C16:1 | 3.22±0.68 | 2.75±1.36 | 3.27±0.74 | 0.11 | 0.14 | 0.98 |
| 10 | lysoPCa C17:0 | 2.11±0.54 | 1.67±0.69 | 2.02±0.48 | < 0.01 | 0.07 | 0.83 |
| 11 | lysoPCa C18:0 | 48.90±10.67 | 34.78±14.32 | 40.63±9.36 | < 0.01 | 0.17 | < 0.05 |
| 12 | lysoPCa C18:1 | 23.01±5.85 | 19.28±8.33 | 22.93±5.04 | < 0.05 | 0.11 | 1.00 |
| 13 | lysoPCa C18:2 | 47.28±15.01 | 38.27±15.01 | 49.58±13.65 | < 0.05 | < 0.05 | 0.83 |
| 14 | lysoPCa C20:3 | 2.71±0.74 | 2.21±1.00 | 2.60±0.90 | < 0.05 | 0.24 | 0.87 |
| 15 | lysoPCa C20:4 | 7.85±2.20 | 6.99±3.04 | 8.36±3.31 | 0.37 | 0.17 | 0.77 |
| 16 | PCaa C28:1 | 1.64±0.40 | 1.27±0.46 | 1.60±0.43 | < 0.01 | < 0.05 | 0.93 |
| 17 | PCaa C30:0 | 2.30±0.86 | 1.93±0.91 | 2.85±1.16 | 0.19 | < 0.01 | 0.08 |
| 18 | PCaa C32:0 | 10.96±2.34 | 10.10±2.66 | 11.94±2.74 | 0.30 | < 0.05 | 0.32 |
| 19 | PCaa C32:1 | 8.01±4.53 | 7.77±4.17 | 9.81±3.40 | 0.97 | 0.17 | 0.24 |
| 20 | PCaa C32:3 | 0.28±0.05 | 0.22±0.07 | 0.31±0.08 | < 0.01 | < 0.01 | 0.33 |
| 21 | PCaa C34:1 | 166.30±43.39 | 164.48±44.79 | 187.54±39.08 | 0.98 | 0.12 | 0.16 |
| 22 | PCaa C34:2 | 423.34±79.46 | 397.71±100.62 | 444.35±116.12 | 0.47 | 0.17 | 0.69 |
| 23 | PCaa C34:3 | 11.73±2.92 | 10.44±4.28 | 13.05±3.96 | 0.28 | < 0.05 | 0.38 |
| 24 | PCaa C34:4 | 1.09±0.35 | 0.82±0.47 | 1.14±0.44 | < 0.05 | < 0.05 | 0.91 |
| 25 | PCaa C36:0 | 3.55±1.10 | 2.27±0.81 | 3.72±0.96 | < 0.01 | < 0.01 | 0.78 |
| 26 | PCaa C36:1 | 38.25±9.16 | 31.79±10.21 | 37.82±8.39 | < 0.01 | < 0.05 | 0.98 |
| 27 | PCaa C36:2 | 236.74±46.67 | 195.88±54.83 | 222.73±61.14 | < 0.01 | 0.15 | 0.58 |
| 28 | PCaa C36:3 | 94.63±20.27 | 82.99±31.65 | 92.22±31.93 | 0.16 | 0.43 | 0.94 |
| 29 | PCaa C36:4 | 160.24±32.77 | 156.51±56.06 | 168.18±54.67 | 0.94 | 0.63 | 0.81 |
| 30 | PCaa C36:5 | 13.55±7.17 | 10.63±4.09 | 13.81±7.87 | 0.11 | 0.15 | 0.99 |
| 31 | PCaa C36:6 | 0.58±0.20 | 0.41±0.22 | 0.66±0.28 | < 0.01 | < 0.01 | 0.44 |
| 32 | PCaa C38:0 | 3.37±0.92 | 2.63±0.81 | 3.64±0.86 | < 0.01 | < 0.01 | 0.46 |
| 33 | PCaa C38:3 | 37.87±10.60 | 31.18±12.91 | 36.26±13.59 | < 0.05 | 0.27 | 0.87 |
| 34 | PCaa C38:4 | 84.43±18.91 | 75.84±26.99 | 81.64±27.36 | 0.26 | 0.64 | 0.90 |
| 35 | PCaa C38:5 | 34.26±8.93 | 30.23±9.45 | 35.31±9.97 | 0.14 | 0.11 | 0.91 |
| 36 | PCaa C38:6 | 80.13±26.35 | 76.46±25.49 | 90.56±29.64 | 0.82 | 0.13 | 0.31 |
| 37 | PCaa C40:2 | 0.42±0.11 | 0.29±0.10 | 0.41±0.09 | < 0.01 | < 0.01 | 0.77 |
| 38 | PCaa C40:3 | 0.58±0.13 | 0.44±0.13 | 0.56±0.14 | < 0.01 | < 0.01 | 0.82 |
| 39 | PCaa C40:4 | 2.89±0.82 | 2.41±0.92 | 2.83±0.91 | < 0.05 | 0.17 | 0.97 |
| 40 | PCaa C40:5 | 8.18±2.58 | 7.05±2.68 | 8.39±2.80 | 0.15 | 0.15 | 0.95 |
| 41 | PCaa C40:6 | 29.18±9.92 | 26.74±9.52 | 31.41±12.62 | 0.56 | 0.22 | 0.70 |
| 42 | PCaa C42:0 | 0.53±0.19 | 0.50±0.17 | 0.72±0.20 | 0.80 | < 0.01 | < 0.01 |
| 43 | PCaa C42:1 | 0.33±0.11 | 0.28±0.10 | 0.40±0.09 | 0.14 | < 0.01 | < 0.05 |
| 44 | PCaa C42:2 | 0.32±0.08 | 0.22±0.07 | 0.30±0.06 | < 0.01 | < 0.01 | 0.61 |
| 45 | PCaa C42:4 | 0.23±0.05 | 0.17±0.06 | 0.23±0.06 | < 0.01 | < 0.01 | 0.80 |
| 46 | PCaa C42:5 | 0.34±0.10 | 0.24±0.10 | 0.35±0.22 | < 0.01 | < 0.01 | 0.91 |
| 47 | PCae C32:1 | 1.72±0.42 | 1.45±0.41 | 2.09±0.55 | < 0.05 | < 0.01 | < 0.01 |
| 48 | PCae C32:2 | 0.45±0.12 | 0.33±0.10 | 0.50±0.13 | < 0.01 | < 0.01 | 0.21 |
| 49 | PCae C34:0 | 1.02±0.25 | 0.83±0.25 | 1.11±0.27 | < 0.01 | < 0.01 | 0.31 |
| 50 | PCae C34:1 | 5.19±1.10 | 4.91±1.27 | 6.11±1.36 | 0.56 | < 0.01 | < 0.05 |
| 51 | PCae C34:2 | 8.99±2.48 | 6.59±2.46 | 9.34±2.78 | < 0.01 | < 0.01 | 0.86 |
| 52 | PCae C34:3 | 6.64±1.98 | 4.08±1.87 | 6.16±2.31 | < 0.01 | < 0.01 | 0.65 |
| 53 | PCae C36:0 | 0.80±0.17 | 0.61±0.14 | 0.82±0.14 | < 0.01 | < 0.01 | 0.78 |
| 54 | PCae C36:1 | 7.44±1.30 | 6.45±1.72 | 7.93±1.64 | < 0.05 | < 0.01 | 0.46 |
| 55 | PCae C36:2 | 10.01±1.94 | 8.85±2.48 | 10.68±2.65 | 0.08 | < 0.05 | 0.53 |
| 56 | PCae C36:3 | 5.67±1.36 | 4.23±1.46 | 5.78±1.64 | < 0.01 | < 0.01 | 0.96 |
| 57 | PCae C36:4 | 14.16±3.63 | 10.92±3.98 | 14.48±4.20 | < 0.01 | < 0.01 | 0.95 |
| 58 | PCae C36:5 | 10.39±2.80 | 7.37±2.59 | 10.79±3.41 | < 0.01 | < 0.01 | 0.86 |
| 59 | PCae C38:0 | 1.43±0.39 | 1.08±0.36 | 1.45±0.42 | < 0.01 | < 0.01 | 0.97 |
| 60 | PCae C38:2 | 1.60±0.45 | 1.24±0.46 | 1.56±0.43 | < 0.01 | < 0.05 | 0.92 |
| 61 | PCae C38:3 | 3.74±0.70 | 3.10±1.10 | 3.87±1.09 | < 0.05 | < 0.01 | 0.86 |
| 62 | PCae C38:4 | 8.75±1.84 | 7.89±2.39 | 9.82±2.55 | 0.20 | < 0.01 | 0.17 |
| 63 | PCae C38:5 | 12.49±2.73 | 10.83±3.11 | 13.51±3.32 | < 0.05 | < 0.01 | 0.41 |
| 64 | PCae C38:6 | 6.72±1.75 | 4.95±1.57 | 7.08±1.51 | < 0.01 | < 0.01 | 0.68 |
| 65 | PCae C40:1 | 1.21±0.30 | 0.90±0.35 | 1.18±0.26 | < 0.01 | < 0.01 | 0.95 |
| 66 | PCae C40:2 | 1.36±0.26 | 1.15±0.32 | 1.52±0.40 | < 0.05 | < 0.01 | 0.15 |
| 67 | PCae C40:3 | 1.17±0.26 | 0.91±0.29 | 1.21±0.26 | < 0.01 | < 0.01 | 0.86 |
| 68 | PCae C40:4 | 1.88±0.40 | 1.58±0.52 | 2.10±0.43 | < 0.05 | < 0.01 | 0.16 |
| 69 | PCae C40:5 | 2.86±0.65 | 2.55±0.65 | 3.23±0.55 | 0.08 | < 0.01 | 0.08 |
| 70 | PCae C40:6 | 3.89±0.95 | 3.45±0.93 | 4.52±0.79 | 0.08 | < 0.01 | < 0.05 |
| 71 | PCae C42:1 | 0.33±0.07 | 0.25±0.09 | 0.30±0.08 | < 0.01 | < 0.05 | 0.32 |
| 72 | PCae C42:2 | 0.48±0.10 | 0.36±0.15 | 0.53±0.18 | < 0.01 | < 0.01 | 0.35 |
| 73 | PCae C42:3 | 0.76±0.17 | 0.59±0.21 | 0.83±0.17 | < 0.01 | < 0.01 | 0.38 |
| 74 | PCae C42:4 | 0.75±0.22 | 0.64±0.26 | 0.92±0.29 | 0.17 | < 0.01 | < 0.05 |
| 75 | PCae C42:5 | 1.55±0.40 | 1.50±0.42 | 1.93±0.45 | 0.89 | < 0.01 | < 0.01 |
| 76 | PCae C44:3 | 0.11±0.02 | 0.09±0.03 | 0.12±0.04 | < 0.01 | < 0.01 | 0.46 |
| 77 | PCae C44:4 | 0.29±0.08 | 0.25±0.08 | 0.35±0.11 | 0.22 | < 0.01 | < 0.05 |
| 78 | PCae C44:5 | 1.21±0.40 | 1.21±0.44 | 1.67±0.58 | 1.00 | < 0.01 | < 0.01 |
| 79 | PCae C44:6 | 1.28±0.45 | 1.21±0.44 | 1.66±0.54 | 0.77 | < 0.01 | < 0.01 |
| 80 | SM(OH) C14:1 | 5.66±1.22 | 4.74±1.49 | 6.10±1.33 | < 0.01 | < 0.01 | 0.44 |
| 81 | SM(OH) C16:1 | 4.28±0.83 | 3.79±1.01 | 4.69±0.85 | 0.05 | < 0.01 | 0.22 |
| 82 | SM(OH) C22:1 | 41.60±8.21 | 30.34±10.67 | 39.37±9.45 | < 0.01 | < 0.01 | 0.65 |
| 83 | SM(OH) C22:2 | 40.38±6.90 | 30.36±9.14 | 40.55±6.99 | < 0.01 | < 0.01 | 1.00 |
| 84 | SM(OH) C24:1 | 1.64±0.36 | 1.22±0.43 | 1.52±0.41 | < 0.01 | < 0.05 | 0.48 |
| 85 | SM C16:0 | 179.41±25.33 | 156.40±34.86 | 189.61±36.47 | < 0.01 | < 0.01 | 0.45 |
| 86 | SM C16:1 | 24.96±3.73 | 21.68±5.40 | 26.32±6.69 | < 0.05 | < 0.01 | 0.58 |
| 87 | SM C18:0 | 43.74±8.01 | 43.00±11.01 | 49.70±11.09 | 0.94 | < 0.05 | 0.07 |
| 88 | SM C18:1 | 19.36±4.05 | 18.70±5.45 | 22.33±5.73 | 0.83 | < 0.05 | 0.07 |
| 89 | SM C20:2 | 0.69±0.22 | 0.67±0.19 | 0.79±0.20 | 0.93 | 0.07 | 0.13 |
| 90 | SM C24:0 | 49.70±9.10 | 36.90±12.33 | 46.41±11.27 | < 0.01 | < 0.01 | 0.49 |
| 91 | SM C24:1 | 173.03±28.21 | 155.29±39.94 | 182.72±32.75 | 0.06 | < 0.01 | 0.53 |
| 92 | SM C26:0 | 0.31±0.08 | 0.24±0.08 | 0.31±0.08 | < 0.01 | < 0.01 | 1.00 |
| 93 | SM C26:1 | 0.57±0.15 | 0.52±0.18 | 0.57±0.18 | 0.30 | 0.49 | 0.99 |
| 94 | Alanine | 409.67±87.56 | 397.41±130.47 | 442.98±90.47 | 0.87 | 0.25 | 0.47 |
| 95 | Arginine | 70.12±27.96 | 72.36±30.69 | 91.96±25.80 | 0.94 | < 0.05 | < 0.05 |
| 96 | Asparagine | 51.64±10.81 | 58.18±13.27 | 63.89±13.32 | 0.06 | 0.20 | < 0.01 |
| 97 | Aspartate | 11.35±2.86 | 9.02±2.90 | 7.43±3.29 | < 0.01 | 0.12 | < 0.01 |
| 98 | Citrulline | 29.07±8.85 | 25.60±8.20 | 24.47±7.41 | 0.16 | 0.87 | 0.10 |
| 99 | Glutamine | 662.06±105.23 | 618.51±114.21 | 658.21±112.99 | 0.20 | 0.38 | 0.99 |
| 100 | Glutamate | 88.11±35.77 | 85.74±40.50 | 64.14±32.62 | 0.96 | 0.08 | < 0.05 |
| 101 | Glycine | 267.73±68.56 | 249.34±99.00 | 305.51±56.86 | 0.57 | < 0.05 | 0.18 |
| 102 | Histidine | 90.58±12.10 | 82.04±17.40 | 92.52±14.85 | < 0.05 | < 0.05 | 0.88 |
| 103 | Isoleucine | 88.41±25.22 | 109.93±37.40 | 84.65±18.65 | < 0.01 | < 0.01 | 0.88 |
| 104 | Leucine | 158.14±42.38 | 184.18±67.03 | 145.80±35.97 | 0.08 | < 0.05 | 0.65 |
| 105 | Lysine | 272.84±67.99 | 302.08±70.91 | 291.57±69.51 | 0.16 | 0.84 | 0.57 |
| 106 | Methionine | 30.12±7.54 | 33.94±8.71 | 33.36±12.39 | 0.17 | 0.97 | 0.39 |
| 107 | Ornithine | 119.64±56.92 | 122.61±51.13 | 109.65±42.24 | 0.97 | 0.62 | 0.75 |
| 108 | Phenylalanine | 70.53±13.13 | 77.93±15.40 | 74.23±14.19 | 0.06 | 0.60 | 0.59 |
| 109 | Proline | 201.86±54.49 | 215.61±68.43 | 229.99±52.27 | 0.57 | 0.64 | 0.18 |
| 110 | Serine | 120.64±19.69 | 125.30±28.56 | 139.96±29.45 | 0.70 | 0.09 | < 0.05 |
| 111 | Threonine | 123.73±26.33 | 137.03±32.24 | 164.85±51.05 | 0.22 | < 0.05 | < 0.01 |
| 112 | Tryptophan | 62.55±12.61 | 60.83±18.08 | 61.51±9.87 | 0.86 | 0.98 | 0.96 |
| 113 | Tyrosine | 69.39±18.49 | 77.57±19.20 | 68.75±13.79 | 0.11 | 0.16 | 0.99 |
| 114 | Valine | 271.45±59.11 | 295.10±85.31 | 263.01±44.50 | 0.28 | 0.19 | 0.89 |
| 115 | Creatinine | 105.04±30.05 | 83.77±23.85 | 90.58±29.15 | < 0.01 | 0.63 | 0.13 |
| 116 | Serotonin | 1.04±0.75 | 0.60±0.63 | 0.69±0.57 | < 0.05 | 0.88 | 0.12 |
| 117 | Taurine | 146.76±44.65 | 88.54±52.30 | 106.44±56.71 | < 0.01 | 0.38 | < 0.01 |

^a^One-way ANOVA with Tukey’s post hoc test and with FDR correction.

Abbreviations: GBS, Guillain-Barre syndrome; MS, multiple sclerosis.

Supplementary Table 2. Receiver operating characteristics (ROC) curves with area under curve (AUC) of single or combined metabolites to discriminate GBS patients from the controls.

| **Metabolites** | **AUC (95% CI)** | **Sensitivity** | **Specificity** |
| --- | --- | --- | --- |
| PCaa C42:2 | 0.83 (0.73-0.92) | 0.76 | 0.80 |
| PCae C36:0 | 0.81 (0.72-0.91) | 0.82 | 0.78 |
| PCaa C40:2 | 0.81 (0.71-0.91) | 0.68 | 0.88 |
| SM C24:0 | 0.79 (0.69-0.90) | 0.76 | 0.80 |
| PCae C40:1 | 0.75 (0.62-0.85) | 0.71 | 0.75 |
| PCae C40:3 | 0.74 (0.62-0.85) | 0.66 | 0.73 |
| PCae C42:3 | 0.72(0.60-0.84) | 0.63 | 0.80 |
| PCae C42:2 | 0.72 (0.60-0.85) | 0.68 | 0.75 |
| PCae C44:3 | 0.70 (0.58-0.82) | 0.71 | 0.70 |
| PCae C34:0 | 0.69 (0.57-0.81) | 0.53 | 0.80 |
| PCae C40:2 | 0.69 (0.56-0.81) | 0.60 | 0.73 |
| PCae C40:4 | 0.64 (0.51-0.76) | 0.55 | 0.78 |
| Combinations |  |  |  |
| All metabolites | 0.80(0.70-0.91) | 0.79 | 0.78 |
| PCaa C42:2+PCae C36:0+SM C24:0 | 0.86(0.77-0.94) | 0.79 | 0.78 |
| PCaa C42:2+PCaa C40:2+SM C24:0 | 0.84(0.75-0.94) | 0.79 | 0.80 |
| PCaa C42:2+PCae C36:0 | 0.84(0.75-0.93) | 0.82 | 0.73 |
| PCaa C42:2+SM C24:0 | 0.84(0.75-0.93) | 0.68 | 0.93 |
| PCaa C42:2+PCae C40:2 | 0.83(0.74-0.92) | 0.76 | 0.75 |

Supplementary Table 3. Six replicates of analysis of one sample in untargeted metabolites analysis.

| **Replicate** | **Glucose** | **Adenine** | **Pyroglutamate** | **Hypoxanthine** | **Creatine** |
| --- | --- | --- | --- | --- | --- |
| sample 1_01 | 103.5 | 35.6 | 12.9 | 27.3 | 13.7 |
| sample 1_02 | 107.4 | 36.1 | 11.2 | 25.7 | 14.3 |
| sample 1_03 | 117.5 | 36.4 | 12.4 | 29.3 | 15.1 |
| sample 1_04 | 105.2 | 37.3 | 10.6 | 28.1 | 15.9 |
| sample 1_05 | 119.7 | 39.5 | 11.5 | 28.5 | 16.1 |
| sample 1_06 | 115.6 | 34.8 | 12.2 | 27.6 | 16.4 |
| Mean | 111.5 | 36.6 | 11.8 | 27.7 | 15.2 |
| SD | 6.9 | 1.6 | 0.8 | 1.2 | 1.1 |
| CV (%) | 6.2 | 4.5 | 7.1 | 4.3 | 7.2 |

CV: Coefficient of Variation

Supplementary Table 4. Coefficient of Variation (CV) of six replicates of analysis of each sample in targeted metabolites analysis.

| **Series** | **Compounds** | **low QC** | **middle QC** | **high QC** |
| --- | --- | --- | --- | --- |
|  |  | **CV (%)** | | |
| 1 | AC0 | 9% | 10% | 3% |
| 2 | AC2 | 9% | 5% | 4% |
| 3 | AC10:1 | 6% | 12% | 7% |
| 4 | AC10:2 | 46% | 44% | 47% |
| 5 | AC14:1 | 8% | 13% | 12% |
| 6 | AC14:2 | 38% | 30% | 33% |
| 7 | AC18:2 | 7% | 3% | 11% |
| 8 | lysoPCa C16:0 | 3% | 4% | 6% |
| 9 | lysoPCa C16:1 | 2% | 4% | 7% |
| 10 | lysoPCa C17:0 | 2% | 6% | 7% |
| 11 | lysoPCa C18:0 | 2% | 5% | 6% |
| 12 | lysoPCa C18:1 | 2% | 5% | 6% |
| 13 | lysoPCa C18:2 | 1% | 4% | 7% |
| 14 | lysoPCa C20:3 | 4% | 4% | 8% |
| 15 | lysoPCa C20:4 | 1% | 4% | 7% |
| 16 | PC aa C28:1 | 3% | 4% | 2% |
| 17 | PC aa C30:0 | 4% | 6% | 4% |
| 18 | PC aa C32:0 | 3% | 4% | 2% |
| 19 | PC aa C32:1 | 6% | 4% | 6% |
| 20 | PC aa C32:3 | 3% | 3% | 4% |
| 21 | PC aa C34:1 | 4% | 4% | 3% |
| 22 | PC aa C34:2 | 3% | 2% | 4% |
| 23 | PC aa C34:3 | 4% | 3% | 3% |
| 24 | PC aa C34:4 | 5% | 4% | 5% |
| 25 | PC aa C36:0 | 10% | 3% | 4% |
| 26 | PC aa C36:1 | 3% | 3% | 3% |
| 27 | PC aa C36:2 | 3% | 3% | 3% |
| 28 | PC aa C36:3 | 4% | 3% | 3% |
| 29 | PC aa C36:4 | 5% | 3% | 3% |
| 30 | PC aa C36:5 | 5% | 3% | 4% |
| 31 | PC aa C36:6 | 6% | 7% | 6% |
| 32 | PC aa C38:0 | 5% | 3% | 8% |
| 33 | PC aa C38:3 | 3% | 4% | 4% |
| 34 | PC aa C38:4 | 4% | 4% | 3% |
| 35 | PC aa C38:5 | 3% | 4% | 3% |
| 36 | PC aa C38:6 | 4% | 5% | 3% |
| 37 | PC aa C40:2 | 14% | 2% | 7% |
| 38 | PC aa C40:3 | 10% | 5% | 7% |
| 39 | PC aa C40:4 | 3% | 6% | 4% |
| 40 | PC aa C40:5 | 4% | 4% | 2% |
| 41 | PC aa C40:6 | 4% | 5% | 3% |
| 42 | PC aa C42:0 | 6% | 3% | 3% |
| 43 | PC aa C42:1 | 10% | 6% | 5% |
| 44 | PC aa C42:2 | 13% | 4% | 5% |
| 45 | PC aa C42:4 | 10% | 5% | 5% |
| 46 | PC aa C42:5 | 6% | 6% | 3% |
| 47 | PC ae C32:1 | 8% | 6% | 3% |
| 48 | PC ae C32:2 | 6% | 3% | 4% |
| 49 | PC ae C34:0 | 4% | 3% | 3% |
| 50 | PC ae C34:1 | 4% | 4% | 4% |
| 51 | PC ae C34:2 | 4% | 4% | 3% |
| 52 | PC ae C34:3 | 5% | 5% | 3% |
| 53 | PC ae C36:0 | 7% | 9% | 6% |
| 54 | PC ae C36:1 | 4% | 3% | 3% |
| 55 | PC ae C36:2 | 3% | 4% | 4% |
| 56 | PC ae C36:3 | 5% | 4% | 4% |
| 57 | PC ae C36:4 | 5% | 6% | 4% |
| 58 | PC ae C36:5 | 5% | 5% | 4% |
| 59 | PC ae C38:0 | 4% | 3% | 5% |
| 60 | PC ae C38:2 | 13% | 5% | 10% |
| 61 | PC ae C38:3 | 3% | 4% | 4% |
| 62 | PC ae C38:4 | 5% | 6% | 5% |
| 63 | PC ae C38:5 | 6% | 5% | 5% |
| 64 | PC ae C38:6 | 5% | 4% | 5% |
| 65 | PC ae C40:1 | 8% | 2% | 6% |
| 66 | PC ae C40:2 | 5% | 3% | 3% |
| 67 | PC ae C40:3 | 5% | 4% | 2% |
| 68 | PC ae C40:4 | 4% | 4% | 4% |
| 69 | PC ae C40:5 | 4% | 3% | 3% |
| 70 | PC ae C40:6 | 4% | 5% | 5% |
| 71 | PC ae C42:1 | 12% | 1% | 4% |
| 72 | PC ae C42:2 | 9% | 4% | 4% |
| 73 | PC ae C42:3 | 8% | 5% | 4% |
| 74 | PC ae C42:4 | 4% | 3% | 4% |
| 75 | PC ae C42:5 | 4% | 4% | 4% |
| 76 | PC ae C44:3 | 11% | 4% | 5% |
| 77 | PC ae C44:4 | 4% | 5% | 2% |
| 78 | PC ae C44:5 | 4% | 3% | 4% |
| 79 | PC ae C44:6 | 5% | 4% | 4% |
| 80 | SM (OH) C14:1 | 2% | 3% | 3% |
| 81 | SM (OH) C16:1 | 2% | 5% | 4% |
| 82 | SM (OH) C22:1 | 4% | 4% | 4% |
| 83 | SM (OH) C22:2 | 3% | 4% | 4% |
| 84 | SM (OH) C24:1 | 3% | 4% | 5% |
| 85 | SM C16:0 | 3% | 3% | 3% |
| 86 | SM C16:1 | 2% | 4% | 3% |
| 87 | SM C18:0 | 2% | 4% | 3% |
| 88 | SM C18:1 | 1% | 4% | 3% |
| 89 | SM C20:2 | 3% | 8% | 6% |
| 90 | SM C24:0 | 2% | 4% | 4% |
| 91 | SM C24:1 | 1% | 5% | 5% |
| 92 | SM C26:0 | 8% | 4% | 7% |
| 93 | SM C26:1 | 3% | 10% | 8% |
| 94 | Alanine | 8% | 2% | 6% |
| 95 | Arginine | 13% | 11% | 8% |
| 96 | Asparagine | 7% | 5% | 7% |
| 97 | Aspartate | 21% | 5% | 10% |
| 98 | Citrulline | 16% | 5% | 6% |
| 99 | Glutamine | 14% | 12% | 8% |
| 100 | Glutamate | 7% | 6% | 8% |
| 101 | Glycine | 13% | 10% | 7% |
| 102 | Histidine | 7% | 6% | 8% |
| 103 | Isoleucine | 7% | 6% | 6% |
| 104 | Leucine | 5% | 8% | 6% |
| 105 | Lysine | 15% | 11% | 13% |
| 106 | Methionine | 16% | 18% | 11% |
| 107 | Ornithine | 19% | 12% | 18% |
| 108 | Phenylalanine | 7% | 5% | 10% |
| 109 | Proline | 6% | 6% | 3% |
| 110 | Serine | 13% | 7% | 9% |
| 111 | Threonine | 9% | 3% | 5% |
| 112 | Tryptophan | 14% | 6% | 7% |
| 113 | Tyrosine | 4% | 6% | 2% |
| 114 | Valine | 8% | 7% | 14% |
| 115 | Creatinine | 17% | 19% | 6% |
| 116 | Serotonin | 182% | 3% | 5% |
| 117 | Taurine | 8% | 3% | 4% |

QC: Quality control


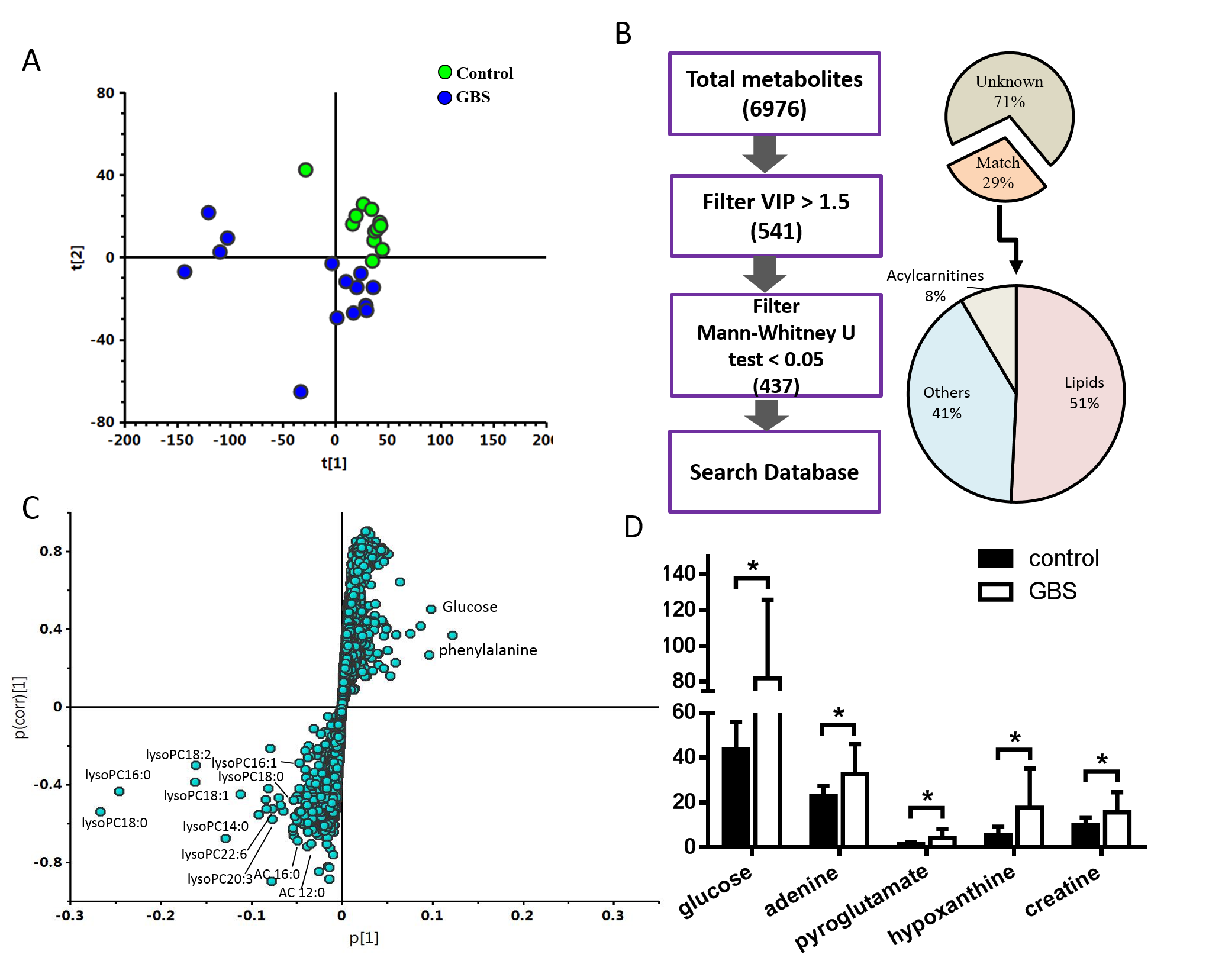


**Supplementary figure S1. Changes in global metabolome of Guillain-Barre syndrome (GBS) patients and healthy controls (control).** (A) Extracted plasma from GBS patients (n = 14) and healthy controls (n = 12) were analyzed by UPLC-TOF/MS in electrospray positive ion mode performed. The principal component analysis demonstrates a clear separation of metabolites between GBS patients (blue) and healthy controls (green). (B) According to the retention characteristics, and mass spectra alignment, 6976 features were picked out. These features were analyzed with VIP score and Student t-test. Four hundred and fifty one features showed high VIP scores (≥ 1.5) and significant differences (p < 0.05) in plasma levels between the GBS patients and the healthy controls. Information about significantly changed metabolites was searched from the HMDB (http://www.hmdb.ca/) database, and 29% features-matched candidates containing lipids (51%), acylcarnitines (8%), and others (41%). (C) Orthogonal partial least squares discriminate analysis (PLS-DA) S-plot of 29% features-matched candidates. This plot shows a binary comparison of GBS patients and controls. Features in the bottom left quadrant indicate higher levels in the controls, and features in the upper right indicate higher levels in GBS patients. (D) Significantly increased metabolites in GBS patients compared to the healthy controls (control). Statistical differences were determined by Student’s two tailed t-test. *p < 0.05.


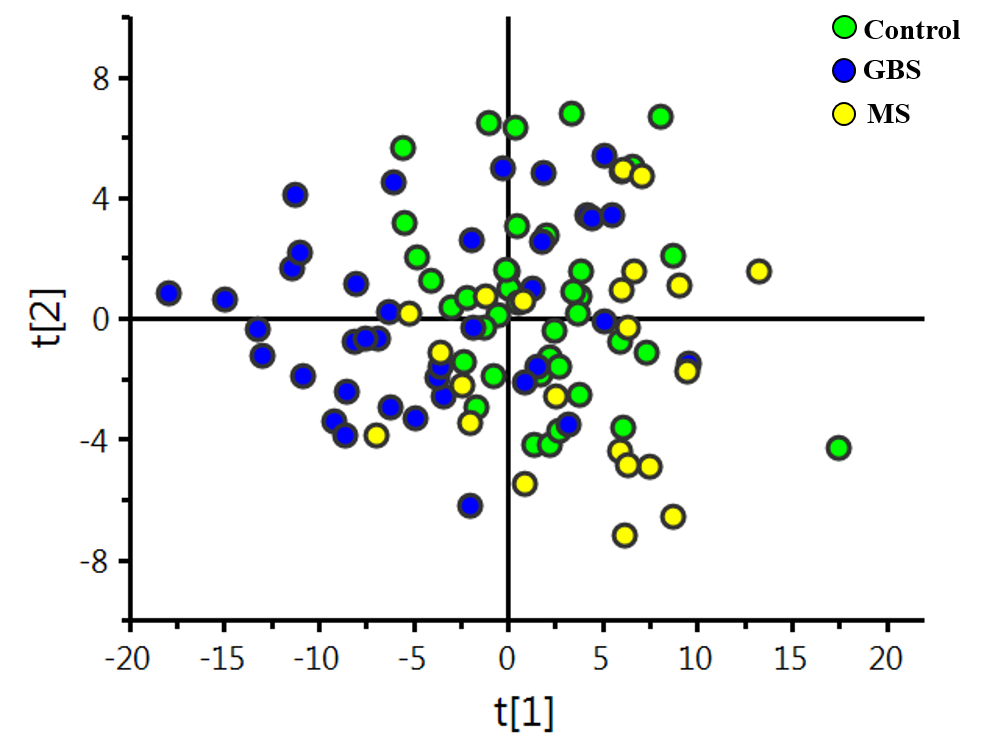


**Supplementary figure S2. Targeted metabolomics profiles in the GBS patients, the multiple sclerosis (MS) patients, and the healthy controls (control).** Plasma from GBS patients (n = 38), MS patients (n = 22) and healthy controls (n = 40) were analyzed by LC-MS/MS and FIA-MS/MS in electrospray positive and negative ion mode. The principal component analysis (PCA) demonstrates a separation of metabolites between GBS, MS, and control cases.


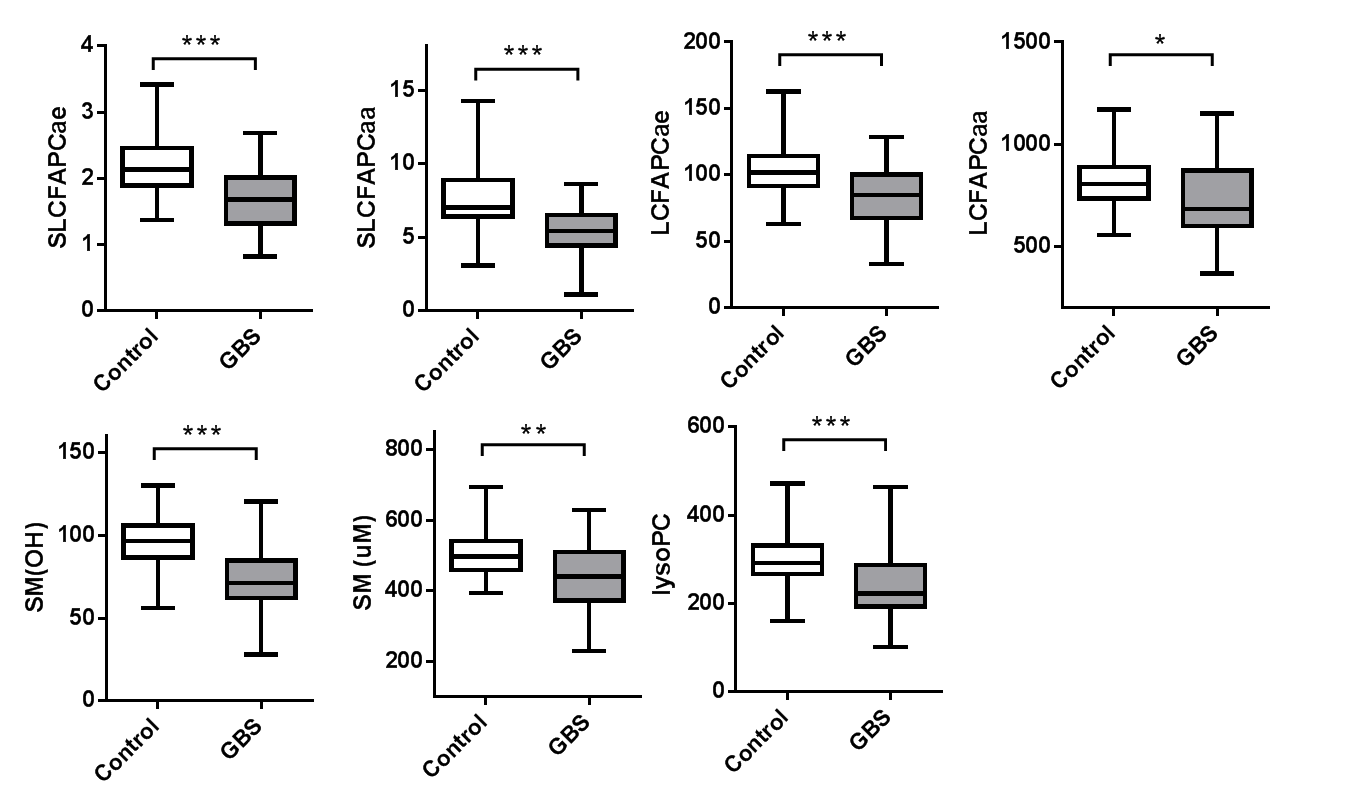


**Supplementary figure S3. Significantly decreased levels of lipophilic metabolites in GBS patients.** The concentrations of LCFA (PCae), LCFA (PCaa), SLCFA (PCae), SLCFA (PCaa), SM, SM(OH), and lysoPC were determined by combining these similar types of metabolites in targeted metabolic analysis. Data were shown as mean ± SD in the control (n = 40) and the GBS (n = 38) groups. Statistical differences were determined by Student’s t-test; * p < 0.05; ** p < 0.01; *** p < 0.001. PCae, phosphatidylcholine acyl-alkyl; LCFA, long chain fatty acid; SLCFA, saturated long chain fatty acid; SM, sphingomyelin; SM(OH), hydroxysphingomyelin; lysoPC, lysophosphatidylcholine.

**Supplementary figure S4. Levels of cholesterol in the GBS patients compared with healthy controls.** Quantitative data were determined by LC-MS/MS. Data were shown as mean ± SD in the controls (n = 15), GBS patients (n = 14), and MS patients (n = 16). Statistical differences were determined by Mann-Whitney U test.

**
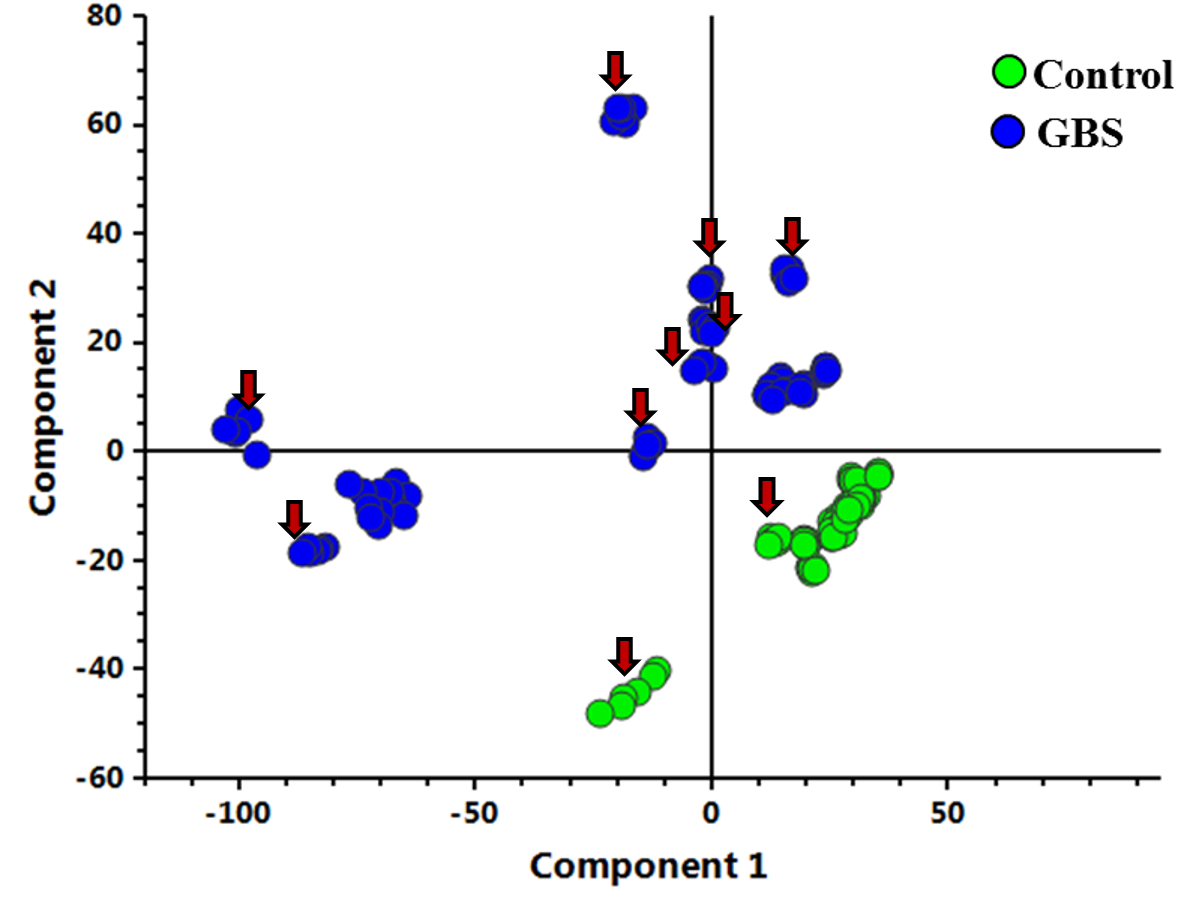
**

**Supplementary figure S5. Quality check in untargeted analysis with UPLC-TOF/MS in electrospray positive ion mode.** Principal component analysis (PCA) showed that six replicates of each selected sample were clustered in their metabolic profile (red arrow), indicating reproducibility of the method.
